# The effect of using isomaltulose (Palatinose™) to modulate the glycaemic properties of breakfast on the cognitive performance of children

**DOI:** 10.1007/s00394-014-0779-8

**Published:** 2014-10-14

**Authors:** Hayley Young, David Benton

**Affiliations:** Department of Psychology, University of Wales Swansea, Swansea, SA2 8PP Wales, UK

**Keywords:** Breakfast, Children, Cognition, Glycaemic load, Mood, Isomaltulose

## Abstract

**Purpose:**

Although previous research has associated the glycaemic load (GL) of a meal with cognitive functioning, typically the macro-nutrient composition of the meals has differed, raising a question as to whether the response was to GL or to the energy, nutrients or particular foods consumed. Therefore, the present study contrasted two breakfasts that offered identical levels of energy and macro-nutrients, although they differed in GL.

**Methods:**

Using a repeated-measures, double-blind design, 75 children aged 5–11 years, from socially deprived backgrounds, attended a school breakfast club and on two occasions, at least a week apart, they consumed a meal sweetened with either isomaltulose (Palatinose™) (GL 31.6) or glucose (GL 59.8). Immediate and delayed verbal memory, spatial memory, sustained attention, reaction times, speed of information processing and mood were assessed 1 and 3 h after eating.

**Results:**

The nature of the meals did not influence any measure of cognition or mood after an hour; however, after 3 h, children’s memory and mood improved after the lower-GL breakfast. If children had eaten the lower-GL meal on the second day of testing, they were able to process information faster and had better spatial memory later in the morning.

**Conclusions:**

Towards the end of a morning in school, having consumed a lower-GL breakfast resulted in better mood and aspects of cognitive functioning.

## Introduction

There have been various reports that memory is influenced adversely by missing breakfast [1, 2] or consuming a small meal [3], effects that can be reversed by a glucose-containing drink [1] or a snack [3]. However, a glucose drink produces a rapid, large and relatively short-term increase that is followed by a rapid fall in blood glucose. When blood glucose levels fall, poorer mood and increased irritability may result [4, 5]. More recently, attempts have been made to consider the macro-nutrient composition of meals in the hope of establishing the optimal nutritional profile. In adults [6, 7], adolescents [8] and children [9, 10], breakfasts that release glucose slowly (low-glycaemic load, GL) are associated with better cognitive functioning in the late morning. Although studies have focused attention on the glycaemic properties of the meals, to date typically this has been achieved by varying the type of food consumed or the nature and amount of various macro-nutrients. Thus, it is unclear whether it is the glycaemic response that is important rather than the macro-nutrient composition of the meal. The aim of the present study was therefore to consider the influence of the GL of meals that are otherwise identical in their macro-nutrient composition.

Although glycaemic index (GI) has been widely used to indicate the speed at which glucose obtained from dietary carbohydrate is released into the blood, it is a measure based on 50 g of available carbohydrate [11]. As such, it measures the nature of the carbohydrate but not the quantity provided by a particular portion size: thus a small portion of a high-GI food may have a smaller impact on blood glucose than a larger portion of a low-GI food. For this reason in the present context, GL rather than GI was used. GL is the GI of the various foods consumed, multiplied by the amount of carbohydrate in grams provided by each food and divided by 100. To vary the GL, while keeping the macro-nutrient composition constant, two meals were created that were identical except one was sweetened with isomaltulose (Palatinose™) and one with glucose. Isomaltulose is a disaccharide made from sucrose by the enzymatic rearrangement of the alpha 1, 2 linkage, between glucose and fructose, to an alpha 1, 6 linkage. It is fully digested and absorbed and provides the same energy as other carbohydrates. Isomaltulose, compared with sucrose or glucose, results in lower postprandial blood glucose and insulin responses over a prolonged period [12]. The GI of isomaltulose is 32, sucrose 65 and glucose 100. Therefore, adding either isomaltulose or glucose to a standard meal allowed the GL to be varied while keeping the macro-nutrient and energy intake constant.

There are isolated studies of the influence of isomaltulose in adults. Kashimura et al. [13] found that both isomaltulose and sucrose helped middle-aged adults to concentrate on a mathematical task. However, although it was claimed that the benefit was greater after isomaltulose, no statistical comparisons were reported. More recently, Dye et al. [14] compared the effects of isomaltulose and sucrose in young adults. Although isomaltulose produced a lower blood glucose profile than sucrose, there were no differences in memory or psychomotor performance. It may be critical that the final testing session took place 115 min after the drink, when greater effects have been reported after 210 min [15], and that the comparison was with sucrose rather than glucose that reduced the difference in GL.

Although the previous findings in adults are unclear, there are reasons to suggest that children may be particularly susceptible to the rate at which glucose is released into the blood. The metabolic rate of the brain increases from birth so that between the ages of 4 and 10 years, it is twice that of the adult [16], that is, a given weight of brain uses double the amount of glucose [16]. Therefore, it may be speculated that a decline in the provision of glucose to a child’s brain, for example 2–3 h following a high-GL meal, may negatively impact on neural functioning. The provision of a lower-GL meal may avoid this consequence. Although a low-GL meal has been reported to have beneficial influences in children [9, 10], only Taib et al. [17] have considered the effects of isomaltulose that was added to growing up milk. In five- to six-year-olds, although focused attention and numerical memory declined across the morning, performance after a drink containing isomaltulose was better. These findings are not, however, easy to interpret given the small dose of isomaltulose used (4.48 g) and the small differences in GL that resulted (14 compared to 20). In addition, after a high-GL glucose drink, spatial memory was better than when isomaltulose had been consumed.

Therefore, the present objective was to consider in children the influence of isomaltulose provided in isocaloric meals with the same macro-nutrient composition, consumed in a dose sufficient to produce a significant difference in GL.

## Methods

### Procedure

After fasting overnight, children attending a school breakfast club were recruited on two different days, at least 1 week apart, and ate one of the two meals designed to differ in their GL. The procedure was double-blind, and the order in which the meals were consumed was randomly generated. Immediately prior to breakfast, the Speed of Information Processing Test was performed although the constraints of the school routine prevented other testing at this stage. Breakfast was eaten between 0815 and 0845 hours, and psychological testing took place twice, between 0900 and 0945 and 1115 and 1200 hours. Between breakfast and testing, the children took part in normal school activities although the policy of having physical education in the afternoon ensured that other than the mid-morning break, these were all class room based. Twice children were removed from the class, and tests were administered on an individual basis in a quiet remote area. Testing always took place in the same order. Initially mood was assessed, followed by immediate memory, speed of information processing, reaction times, the ability to sustain attention and delayed memory. Mostly the battery of tests was chosen because a previous study had found that they were influenced, in children, by the glycaemic properties of a meal [9].

### Subjects

Seventy-five children, aged 5–11 years, average age 8 years 8 months, were recruited from four schools. There were 28 boys and 47 girls in the sample. Their parents reported that participants were in good health and had never experienced an adverse reaction to food. Parents gave their consent to the child taking part, and the study was approved by the local ethics committee and was performed in accordance with the ethical standards laid down in the 1964 Declaration of Helsinki. The procedure was approved by the Psychology Ethics Committee of Swansea University (Psy 07-01). The Welsh Index of Multiple Deprivation (WIMD) describes local areas in terms of a range of indices. Parents provided a postcode from which an index was retrieved relating to their degree of deprivation. The sample came from amongst the most socially deprived areas of Wales. All children were a healthy weight for their height and age. Children were excluded if their parents reported that they had ever experienced a negative reaction to food or they were diabetic. As all children attended a school breakfast club, a meal was habitually eaten at this time of day and consisted of a bowl of cereal with milk, a slice of toast and orange/apple juice. Thus, the children’s usual breakfast was one similar to that provided in the study.

### Meals

The details of the meals are given in Table 1. They were designed to offer a similar intake of energy and macro-nutrients, while differing in GL. The meals were of identical appearance. Children were asked to eat as much as possible of the food provided but were not forced to consume more than they wished. When the child had finished eating any food, remaining was weighed, allowing estimates to be made of the nutritional composition of the meals to be established using food tables [18] and information from manufacturers. In practice, the children ate the majority of their breakfast such that the small amounts left were of no practical significance. After breakfast, children attended class as usual and were asked not to eat or drink (except water) for the remainder of the morning. In the event, ten children did report consuming a snack, such as an apple, and the initial statistical analysis proceeded ‘pro-protocol’.

**Table 1:** Macro-nutrient content of the experimental meals

	Higher GL						Lower GL					
	Kcal	CHO	Pro	Fat	GI	GL	Kcal	CHO	Pro	Fat	GI	GL
Cornflakes 20 g	71	17.6	1.6	0.15	81	14.26	71	17.6	1.6	0.15	81	14.26
Milk semi-skimmed 100 ml	46	5.0	3.3	1.5	21	1.05	46	5.0	3.3	1.5	21	1.05
Glucose 5 g	20	5.0	0	0	100	5.00	–	–	–	–	–	–
Palatinose 5 g	–	–	–	–	–	–	20	5.0	0	0	32	1.60
Low-calorie yoghurt 100 g	41	6.0	4.3	0.25	19	1.14	41	6.0	4.3	0.25	19	1.14
Glucose-sweetened fruit 20 g	95	23.7	0	0	94.5	22.40	–	–	–	–	–	–
Palatinose in sweetened fruit 20 g	–	–	–	–	–	–	95	23.7	0	0	35.7	8.46
Orange drink 200 ml (glucose 16 g)	64	16	0	0	100	16.00	–	–	–	–	–	–
Orange drink 200 ml (isomaltulose 16 g)	–	–	–	–	–	–	64	16	0	0	32	5.12
Total	337	73.3	9.2	1.9	–	59.85	337	73.3	9.2	1.9	–	31.63

*GI* glycaemic index, *GL* glycaemic load

### Memory

The approach was based on the Recall of Objects test of the British Ability Scale [19] that is suggested to be suitable for testing those from 4 to 17.11 years. For 40 s, the child viewed a card with pictures of 20 objects, after which they had 60 s to recall as many objects as possible. On a second and third occasion, the child viewed the card for a further 20 s and had 40 s to recall the items. The numbers of items recalled on the three occasions were added and are reported as the immediate memory score. After the third trial, spatial memory was assessed by placing a blank grid in front of the child and asking them to put a series of pictures in the place on the grid that corresponded to the original card. Delayed memory was assessed after performing the other tests, about 20 min after the initial memory test. The child was again asked to verbally recall as many pictures as possible. When the blank grid was again presented, the child tried for a second time to recall the position of the 20 objects. In total, four sets of objectives were presented, a different version on each of the four occasions the test was completed.

### Speed of information processing

The test is taken from the British Ability Scale [19] and measures how quickly simple mental operations can be performed: it is suitable for those from 5 to 17.11 years. A row of circles was presented, each containing a number of small boxes. The task involved marking the circle containing the most boxes. On each of the six pages, there were five lines of circles. The time taken to complete each of the six pages was recorded. Six comparable forms of the test were used.

### Reaction times

An auditory warning sounded and at the same time a light illuminated and a millisecond timer started. For ten trials, the child as quickly as possible pressed a button that extinguished the light and stopped the timer. The reaction in milliseconds was recorded.

### Ability to sustain attention

The paradigm of Shakow [20] was used. An auditory warning sounded, and, after a delay of either 3 or 12 s, a light illuminated and a millisecond timer started. When the child saw the light, a button was pressed that extinguished the light and stopped the timer. The test consisted of four blocks of six trials. The first and third block of trials had a delay of 3 s, and the second and fourth block had a delay of 12 s. The delay ensures that it is the ability to sustain attention that is measured rather than reaction times.

Initial examination of the data found that, on occasions, there were very long delays before responding. Thus, the mean response times did not reflect the data as they were distorted by a few extreme values. It was decided to look at the instance of long response times, that is, the incidence of lapses in attention. The median response time was about 600 ms when there was a 3 s delay before the light illuminated. Therefore, the number of trials in the bottom quintile of the distribution, those longer than 800 ms, is reported as the number of lapses of attention. The median response time for the 12-s delay was about 750 ms. Those responses in the bottom quintile, that is more than 1,000 ms, were again distinguished and reported as lapses in attention.

### Mood

Children were asked how they felt ‘at this moment’ using an eight-point scale of smiley faces that ranged from very unhappy to very happy. A higher score reflects a better mood.

### Statistical analysis

The data were analysed using appropriate analysis of variance designs, for example, type of meal (glucose/isomaltulose) × 1/3 h after eating × immediate/delayed recall × order of testing (was glucose/isomaltulose consumed on the first or second day of testing). The last variable was a between-subjects factor and the others within-subjects factors. It was also considered whether age, gender or social deprivation moderated the effects of glycaemic load on cognition. Gender was entered into the analysis as an additional between-subjects factor, and age and deprivation were modelled as covariates. To prevent the loss of power associated with the addition of covariates to repeated-measures designs [21], both age and deprivation were mean centred prior to analysis. Where significant interactions resulted, they were further analysed using Bonferroni post hoc tests. Speed of information processing was measured at baseline, and in this analysis, baseline performance was also entered as a covariate. Where in the results section higher-order interactions are not mentioned it should be assumed that they were non-significant.

The basic analysis of variance was carried out ‘pro-protocol’, excluding those children who said that they had snacked (*N* = 10), to maintain the integrity of the study that was designed to contrast two meals with identical macro-nutrient compositions. However, in addition, an ‘intention to treat’ analysis was performed that was identical except that it used all subjects, to exclude the possibility that a systematic bias had been introduced by excluding those who subsequently snacked. In practice, the response was similar in both the main and the ‘intention to treat’ analysis so the data presented are for those who did not eat after breakfast.

## Results

### Speed of information processing

**Fig. 1:**
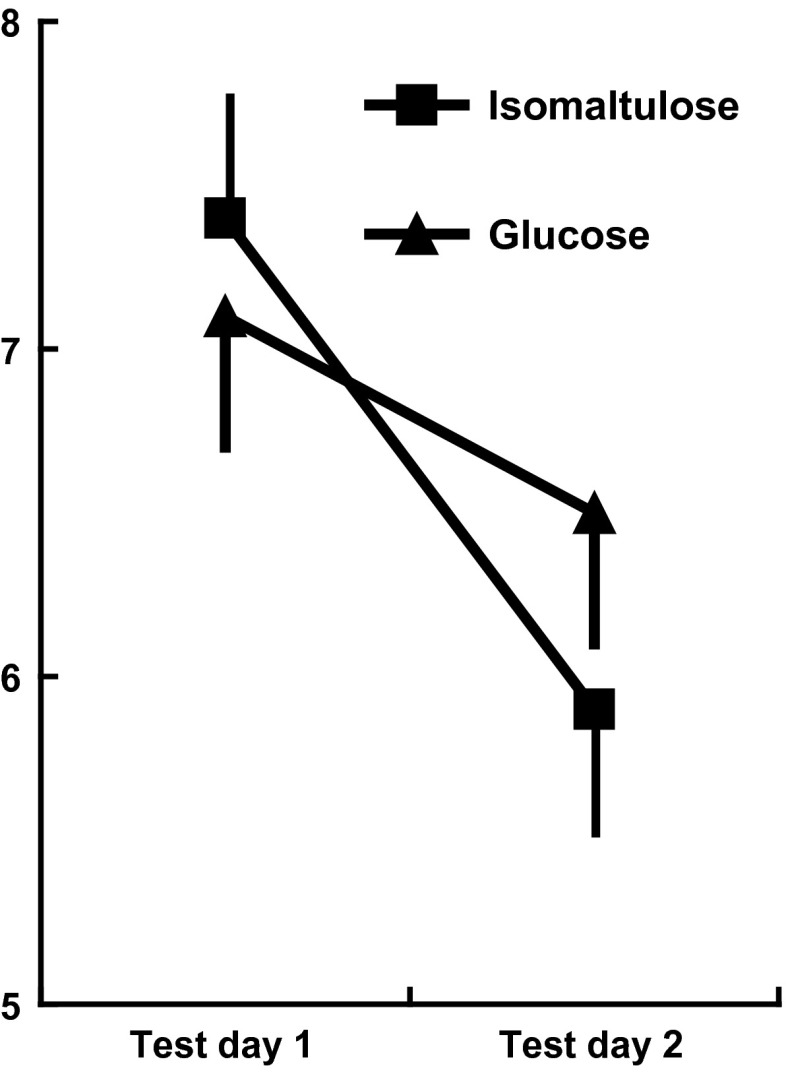
Speed of information processing depending on whether the tests were taken on a first or second occasion. The data are the times taken in seconds ± standard error. On the first day of testing, those eating the two meals did not differ but when tested on a second occasion, those who had eaten the lower-GL meal were significantly quicker (*p* < 0.01)

After breakfast, although the type of meal × 1/3 h after breakfast interaction was non-significant (*F*(1,59) = 0.51, n.s.), the type of meal × order of testing interaction reached significance (*F*(1,59) = 32.56, *p* < 0.0001). The nature of the interaction is illustrated in Fig. 1. There was a general improvement in performance from day one to day two (*p* < 0.0001). There were, however, no significant differences in speed in those eating the two meals when tested on the first day of testing, although the consumption of the isomaltulose (Palatinose™) rather than glucose-based meal was associated with faster performance when tested after consuming a meal for a second time (*p* < 0.01). Neither age, gender nor deprivation moderated the effects of meal on speed of information processing, however, there were main effects of age (*F*(1,59) = 50.42, *p* < 0.0001) and gender (*F*(1,59) = 5.90, *p* < 0.01); older children responded more quickly than younger children (*p* < 0.005) and girls performed better than boys (*p* < 0.002).

### Memory

**Fig. 2:**
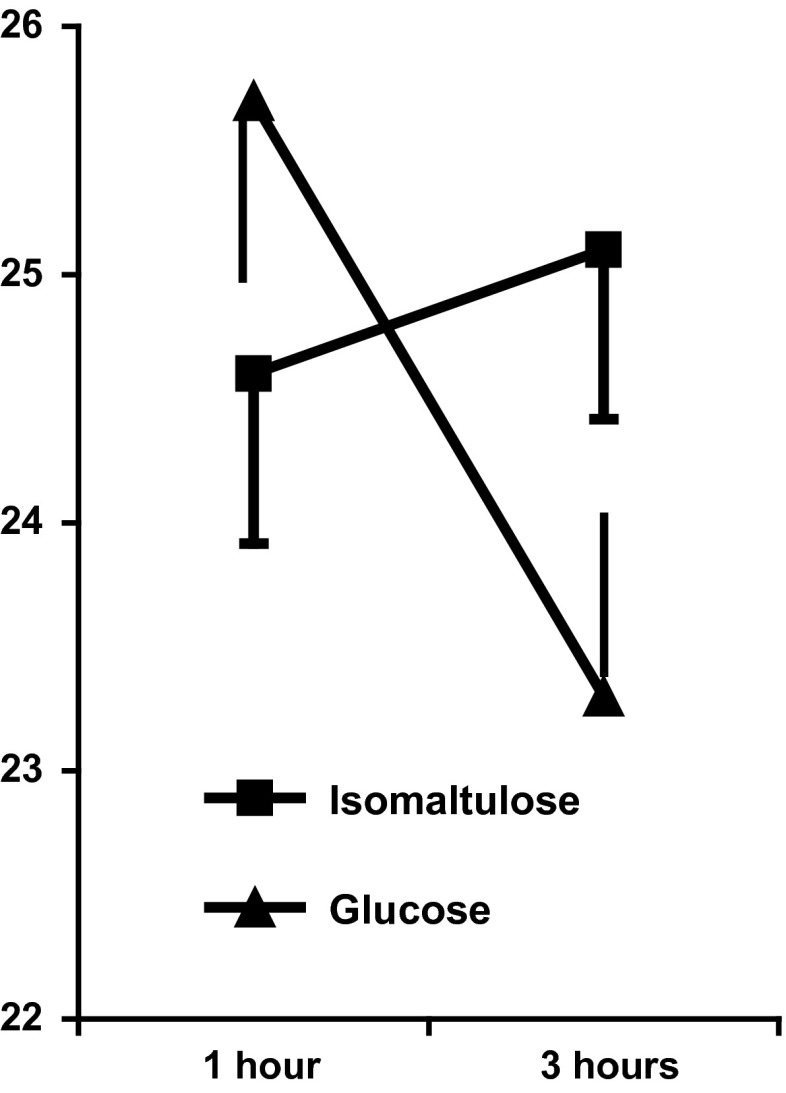
Immediate memory 1 and 3 h after breakfast. The data are mean number of words recalled ± standard error. The two breakfasts did not differ after 1 h, but after 3 h, those eating the lower GL had significantly better memories (*p* < 0.01). The memory significantly declined in those eating the higher (*p* < 0.001) but not lower-GL meal

The interaction type of meal × 1/3 h after breakfast reached statistical significance (*F*(1,69) = 10.501, *p* < 0.002), although the interaction type of meal × 1/3 h after eating × order of testing was non-significant (*F*(1,69) = 0.44, n.s.). Figure 2 illustrates the interaction. When memory was assessed 1 h after breakfast, it was similar in the two groups. However, 3 h after breakfast, those who consumed isomaltulose (Palatinose™) had better memories (*p* < 0.01). Also after eating the glucose-based meal, memory was significantly poorer 3 rather than 1 h following consumption (*p* < 0.001), that is, it declined over the morning. However, after eating the isomaltulose (Palatinose™)-based meal, memory was similar, regardless of whether it was tested 1 or 3 h after breakfast, in fact there was a trend for it to be slightly better later in the morning (Fig. 2). Although there were main effects of age (*F*(1,69) = 31.03, *p* < 0.0001) and deprivation (*F*(1,69) = 3.731, *p* < 0.05), neither factor moderated the effect of meal on memory.

### Spatial memory

**Fig. 3:**
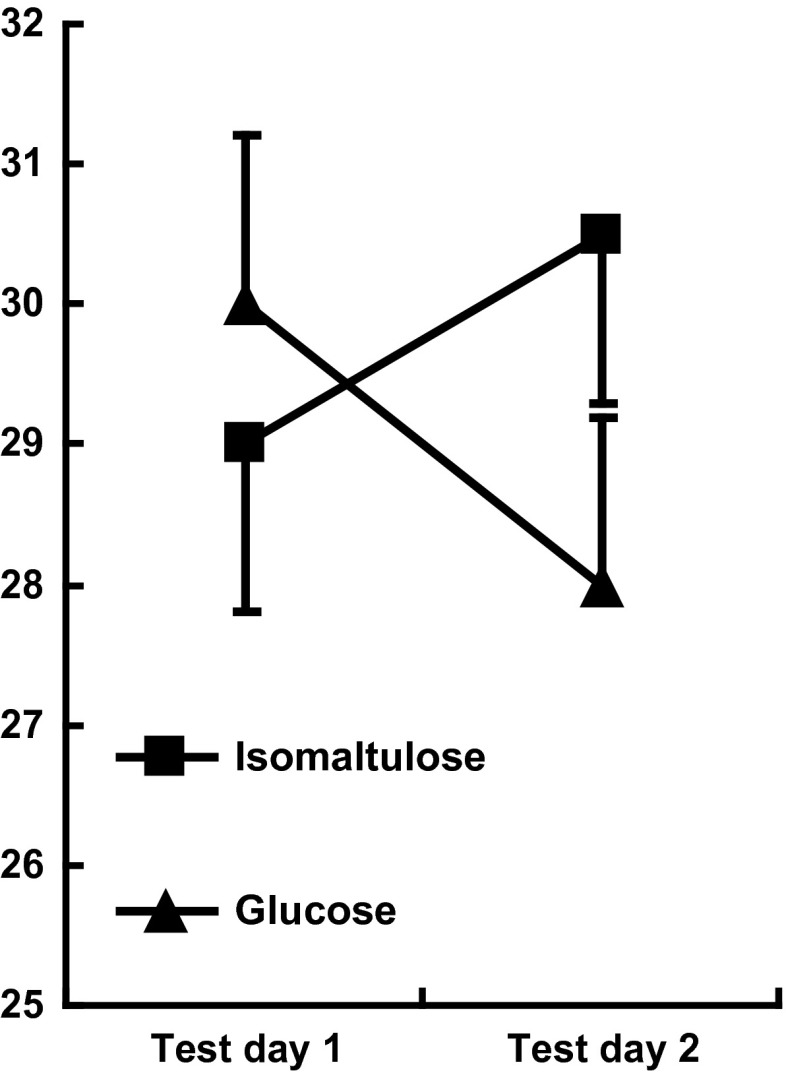
Spatial memory depending on whether the test was taken on the first or second occasion. The data are mean number of correct placements ± standard error. After eating, the lower-GL meal memory was significantly better on the second (*p* < 0.03) but not first day of testing

With spatial memory, the type of meal × 1/3 h after testing (*F*(1,62) = 0.24, n.s) was non-significant. However, the type of meal × order of testing reached statistical significance (*F*(1,69) = 4.19, *p* < 0.04). Post hoc tests showed that on the second test day, in children who had consumed the isomaltulose rather than glucose meal, spatial memory was better (*p* < 0.03; Fig. 3), although the meal consumed did not influence spatial memory on the first day of testing. Gender (*F*(1,69) = 10.80, *p* < 0.001) and age (*F*(1,69) = 8.39, *p* < 0.005) predicted spatial memory, but these effects were independent of the meal consumed.

### Reaction times

Although age predicted reaction times (*F*(1,67) = 56.11, *p* < 0.0001), there were no significant differences between meals or changes over time (*F*(1,67) = 1.16, n.s.). The type of meal × order of testing interaction (*F*(1,67) = 2.08, n.s) was also non-significant.

### Ability to sustain attention

#### Three-second delay

Overall the number of lapses in attention was not influenced by the type of meal eaten (type of meal × 1/3 h after breakfast (*F*(1,67) = 0.02, n.s.); however, the type of meal × time after breakfast × order of testing (*F*(1,67) = 8.85, *p* < 0.004) interaction reached significance. Although the number of lapses of attention was greater 3 rather than 1 h after breakfast (*p* < 0.03), the meal consumed did not influence the number of lapses on the first day of testing. However, on the second test day, those who had consumed the isomaltulose rather than the glucose-based meal tended to have fewer lapses of attention although post hoc tests did not achieve statistical significance. Although age (*F*(1,67) = 36.68, *p* < 0.0001) and social deprivation (*F*(1,67) = 4.77, *p* < 0.03) predicted attention, they did not influence children’s response to breakfast.

#### Twelve-second delay

When the lapses in attention associated with the longer delay were considered, although the type of meal × 1/3 h after breakfast interaction (*F*(1,67) = 0.19, n.s.) was non-significant, the interaction type of meal × time after breakfast × order of testing (*F*(1,67) = 4.46, *p* < 0.04) reached significance. On the first day, the number of lapses of attention was greater 3 rather than 1 h after breakfast (*p* < 0.04), although the type of breakfast consumed was not influential. However, when tested on the second occasion, there was again a trend for those consuming the isomaltulose-based meal to have fewer lapses of attention although no post hoc reached statistical significance. The number of lapses of attention decreased with age (*F*(2,56) = 14.84, *p* < 0.001), but again age did not influence the effect of breakfast.

#### Mood

**Fig. 4:**
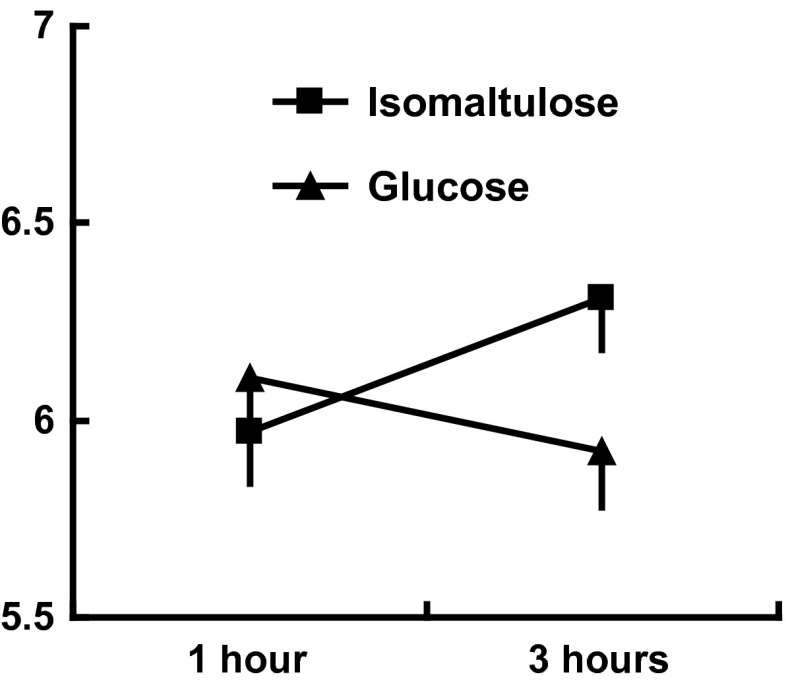
Mood 1 and 3 h after eating. The data are mean mood scores ± standard error where a higher score indicates better mood. There were no significant differences after 1 h although mood was significantly better 3 h after isomaltulose consumption

The interaction type of meal × time reached significance (*F*(1,64) = 5.67, *p* < 0.02). Eating a glucose rather than isomaltulose meal resulted in poorer mood after 3 h (*p* < 0.03), although mood did not differ after 1 h (Fig. 4). Gender predicted mood (*F*(1,64) = 5.17, *p* < 0.02) but did not interact with the nature of the meal.

## Discussion

Previously, studies have varied the breakfasts of children and interpreted beneficial influences on cognition as reflecting the glycaemic properties of the meals. For example, on different days, Ingwersen et al. [10] gave children two breakfast cereals that differed in their speed of absorption (GL 15 vs 27). The lower-GL breakfast was associated with a slower decline in memory and attention throughout the morning. Although the explanation offered was a difference in the glycaemic properties, the possibility cannot be excluded that there were other differences in the food items compared. Similarly, Benton et al. [9] found in children aged 5–8 years that memory, attention and the time spent on task were better in those consuming a meal with a GL of 6 rather than 15 or 39, although the meals had a different macronutrient composition. Thus, whereas any positive outcomes that occurred in this area of study have been interpreted as reflecting differences in GL, differences in the actual foods consumed, the energy provided and in the macro-nutrient composition, prevented the conclusion that GL was necessarily the mechanism. The objective of the present study was to establish whether the response in studies in this area is to GL rather than other aspects of the foods that were contrasted. The present study reported benefits on days when a lower-GL meal was consumed, even though the energy provided and the macro-nutrient composition were identical. As such, it is reasonable to conclude that the response was to differences in the glycaemic properties of the meal.

Although the isomaltulose-based meal benefitted memory (Fig. 2) and mood (Fig. 4), regardless of the day on which it was consumed, there were also effects observed only on the second day of testing. On the second day, children processed information more quickly and had better spatial memory later in the morning after consuming the low-GL breakfast. These observations suggest possible mechanisms by which isomaltulose may be influential as it cannot be assumed that those taking a test battery will be similar on a second rather than first occasion. When first tested the situation is novel and attention grapping; thus, motivation is likely to be greater. However, on later occasions, the tests are familiar and may be viewed as boring, resulting in decreased motivation. The effect on mood in the present study is consistent with such an analysis: isomaltulose resulted in children reporting feeling happier (Fig. 4). Thus, it is possible that on day one, when children were exposed to a novel and interesting situation, they were able to overcome the presumed physiological disadvantage resulting from glucose-based meals. However, by day two when they had become accustomed to the monotonous nature of the tasks, and were likely to be getting a little bored, the lower-GL meal was helpful. Therefore, a hypothesis for further study is that the benefit of a lower-GL meal is that it helps an individual persevere with an uninteresting task. If isomaltulose acts by improving mood and motivation, with a resulting better attitude, then potentially it may have a greater and longer impact. In contrast an effect that lasts for only for one day of testing will have little practical importance. This is of course speculative, and it remains to be demonstrated that the present effects last longer than two days. It is noteworthy that although the use of a crossover design is common when investigating the effects of nutrition on cognition, few studies include the order that meals are consumed in the analysis (e.g. [6, 8, 10, 17, 22]). As such, these findings are difficult to interpret and significant effects on either the first or second testing occasion may have been hidden. For example, Nilsson et al. [23], when they compared the influence of sipping rather than consuming glucose as a bolus of glucose, found that the order that subjects were subject to a condition influenced the effects on working memory task. A consideration of differences in response, depending on prior experience of testing, may suggest underlying mechanisms. Similar to the present study, in young adults, there is a report that that memory was better after drinking glucose, rather than a placebo, but only on the second day of testing [24]. Benton et al. [9] found that a child’s reaction to frustration only improved after the lower-GL breakfast on the first day of testing, perhaps because when faced on a second occasion with a task that was impossible they did not engage.

When comparing studies, the developmental stage of the children may be an important consideration. The present study, that recruited children aged 5–11 years, found that age did not modulate the effect of GL on cognition. That is all children in this age range benefited from a lower GL, although it may be important that during this age range, the demand for brain glucose is at its height and that it begins to decline in the early teenage years. Given that children of the age presently tested have a higher rate of brain glucose utilisation than adults [16], they may be particularly vulnerable to fluctuations in peripheral glucose levels and hence GL: an interesting hypothesis for future study.

Only a few studies have examined the cognitive effects of varying the GL of older children. In 11- to 14-year-olds, Micha et al. [25] investigated the effects of four meals differing in their glycaemic properties, varying both the GI and GL of meals in a systematic way. They found that the greatest facilitation of learning resulted from a low-GI (GI = 48)/high-GL (GL = 41) breakfast, such that both the quantity and nature of carbohydrate were important. However, an important consideration was that the meals that provided a low GL were much smaller (275 and 281 kcal) than those that provided a high GL (469 and 468 kcal). Given that the physical size and caloric intake of breakfast may influence cognition [3], the underlying mechanism was unclear. In addition, because the macronutrient composition of the meals in Micha et al.’s [25] study differed, the response may not have been to GL. In adolescents, Cooper et al. [8] compared a high-GL breakfast (cornflakes and white bread, GL = 54) and a low-GL breakfast (muesli, apple, GL = 36), that although they supplied the same amount of carbohydrate differed in the levels of other macro-nutrients. Nonetheless, the decline across the morning in reaction times and various measures of cognition was less after the lower-GL meal. There are, however, inconsistencies that need to be considered as not all studies have reported a beneficial response to varying the GL of breakfast. In 10- to 12-year-old children, Brindal et al. [26] replaced carbohydrate with protein (GL 18, 24, 33) and failed to find changes in cognitive functioning over a 3-h period. Similarly, Dye et al. [14] examined the effects of 50 g isomaltulose, 50 g sucrose or water and found no effects on measures of cognitive performance in a group of young adults.

There are a number of explanations that may account for inconsistencies in the literature, at least concerning isomaltulose. The present study compared isomaltulose with glucose rather than sucrose, producing a relatively large difference in GL (31.6 vs 59.8), whereas both Dye et al. [14] and Kashimura et al. [13] compared isomaltulose to sucrose resulting in smaller GL differences (GL 16 vs 32; 12.8 vs 26). It may be that larger differences in GL are needed to influence performance. In addition, Dye et al. [14] used a milk-based vehicle and milk is known to increase insulin secretion that both will decrease blood glucose levels and insulin itself is known to modulate cognition [27].

In summary, a low-GI breakfast maintained a child’s performance throughout the morning, particularly in terms of memory and mood. Furthermore, the benefits were achieved by varying the GL of breakfast while maintaining an identical macro-nutrient composition of the meal, allowing the conclusion that the glycaemic nature of the meals was the underlying mechanism. If future studies consider a dose–response curve, this would increase confidence in the phenomenon: to date, this question has not been addressed.
